# Learning Yeast Genetics from Miro Radman

**DOI:** 10.3390/cells10040945

**Published:** 2021-04-20

**Authors:** James E. Haber

**Affiliations:** Department of Biology, Rosenstiel Basic Medical Sciences Research Center, Brandeis University, Waltham, MA 02454-9110, USA; haber@brandeis.edu

**Keywords:** *Saccharomyces cerevisiae*, budding yeast, mismatch repair, DNA damage response, SOS, homologous recombination, heteroduplex rejection

## Abstract

Miroslav Radman’s far-sighted ideas have penetrated many aspects of our study of the repair of broken eukaryotic chromosomes. For over 35 years my lab has studied different aspects of the repair of chromosomal breaks in the budding yeast, *Saccharomyces cerevisiae*. From the start, we have made what we thought were novel observations that turned out to have been predicted by Miro’s extraordinary work in the bacterium *Escherichia coli* and then later in the radiation-resistant *Dienococcus radiodurans*. In some cases, we have been able to extend some of his ideas a bit further.

## 1. When Mismatch Repair Makes the Situation Worse

One of Miro’s stunning discoveries was that *dam recA* double mutants were extremely sensitive to base-pair substitution mutagens such as 2-amino purine (2-AP) [1]. These strains lack the deoxyadenine methyltransferase, which modifies DNA at GATC sites and directs mismatch repair to correct the newly copied DNA strand, and also lack homologous recombination mediated by the RecA protein. In fact, when treated with high doses of 2-AP, these double mutants could produce cells lacking any visible DNA at all [2,3]. However, this mismatch-stimulated killing was blocked by mutations in MutS, MutL, and MutH. The explanation was that—without the hemimethylation of GATC to direct the mismatch repair machinery to the newly replicated strand—MutS/L/H could initiate nicks on either strand and then two 5′ to 3′ exonucleases could converge, producing a double-strand break that required RecA for its repair.

**Figure 1:**
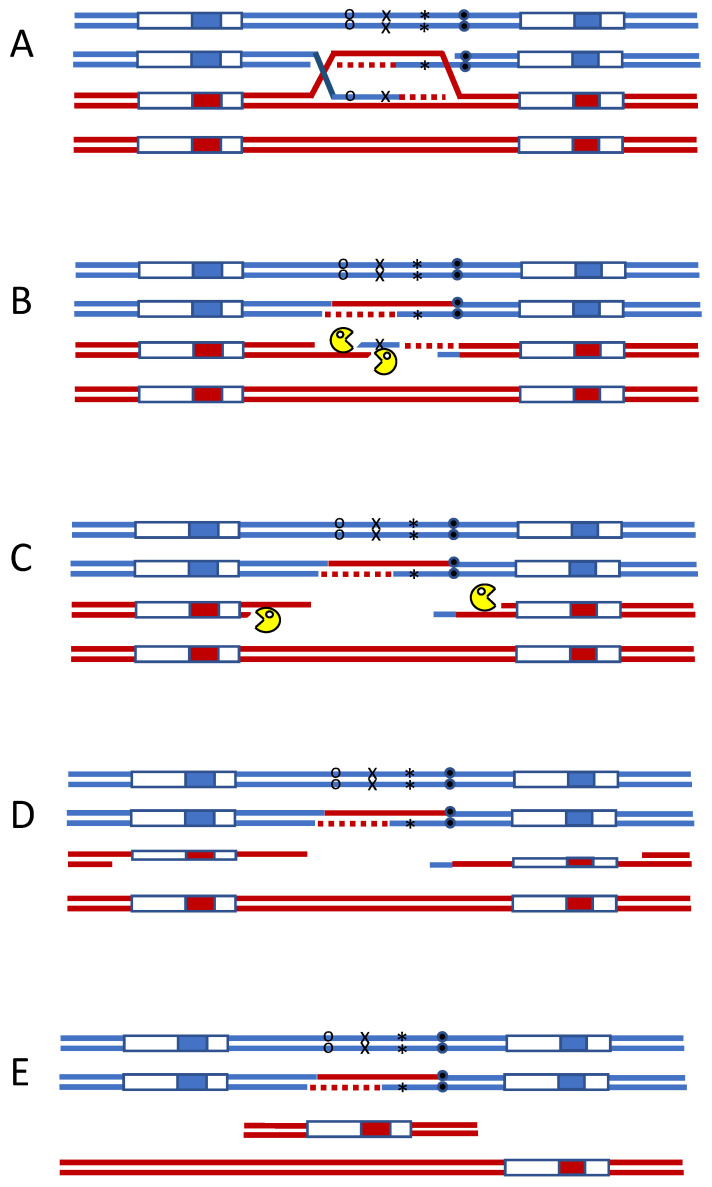
Mismatch repair initiates secondary DSBs during meiotic recombination. Heteroduplex DNA formed during meiotic recombination. (**A**) can be attacked by two independent mismatch repair complexes, oriented on opposite strands (**B**). Exonuclease-dependent removal of adjacent DNA can lead to secondary DSBs (**C**). When the region is flanked by repeated sequences, single-strand annealing (SSA) will result in the deletion of the intervening DNA (**D**,**E**).

The pioneering work of Bob Mortimer and Sy Fogel [4] had defined the key elements of gene conversion and mismatch repair in budding yeast meiosis. In the early-1980s there were no mismatch repair mutations yet identified in yeast, although alleles of some biosynthetic genes showed post-meiotic segregation (PMS), suggesting that they had avoided mismatch repair of heteroduplex DNA during meiotic recombination. At this time, my lab had begun to study meiotic recombination and figured out how to monitor meiotic recombination in real-time using Southern blots [5]. We realized we could also determine the length of gene conversion tracts by inserting a small number of sequence alterations into the homologous DNA segment that we were monitoring [6,7]. The region was created by integrating a pBR322 plasmid containing both a *URA3* gene and a *MAT* sequence into the *MAT* locus, thus creating *MAT***a**------*URA3*---*MAT***a** and *MAT*α-----*URA3*----*MAT*α regions, such that a crossover would produce *MAT***a**-----*URA3*---*MAT*α and *MAT*α-----*URA3*---*MAT***a** reciprocal recombinants that were easily identified because they were non-mating (Figure 1). We introduced about 1 mismatched site each 1 kb along the 9-kb region so that we could estimate gene conversion tract lengths associated with crossing-over. Crossovers occurred in approximately 24% of tetrads, with an average gene conversion tract length of 1.5 kb. What we did not expect were tetrads in which one segregant was Ura3^−^ *MAT***a** or *MAT*α and the other 3 segregants were Ura3^+^, one of which had apparently undergone a crossover and was thus non-mating. Further analysis revealed that the Ura3^−^ segregants had lost *URA3* and the pBR322 sequences and had only a single copy of *MAT***a** or *MAT*α (Figure 1). These exceptional segregants were generally not the result of an unequal crossover that would have created a segregant with three *MAT* loci and a duplication of pBR322 segments. We suggested that these exceptions could arise if crossing-over had occurred in the interval between the flanking *MAT* loci, creating heteroduplex that was subject to mismatch repair [6]. However, if mismatch repair initiated at two separate sites and then two exonucleases, on opposite strands, resected toward each other, they could produce a secondary DSB that could be most easily repaired by single-strand annealing between the flanking *MAT* loci. In our initial paper [5] we made this proposal in complete ignorance of Glickman and Radman’s [1] paper (and also similar results from McGraw and Marinus [8]). By 1990, the first yeast mismatch repair genes, *PMS1*, had been gene cloned and studied [9]; in collaboration with Seymour Fogel’s lab, we showed that knocking out *PMS1* resulted in a suppression of the mismatch-dependent deletions arising in meiosis [10]. By then we had come to appreciate and properly cite Miro’s important insight. In meiosis, recombination between two homologous chromosomes occurs long after replication has been completed, so there are no strand-specific signals that would confine mismatch repair to one or the other chromatid.

All of these results led us to what we called the “Borts uncertainty principle”: To study the details of crossing-over in meiosis we had to introduce heterologies; but those heterologies altered the outcomes.

## 2. A Tale of Rejection

Recombination between mismatched DNA is suppressed, as Miro’s lab spectacularly demonstrated for the case of exchanges between *Escherichia coli* and *Salmonella thyphimurium*, whose genomes are 20% divergent [11,12,13]. This barrier can be overcome by removing the MutS/L/H mismatch repair proteins [11,14]. Later work suggested there might be two pathways to defeat homeologous recombination. One was independent of the MutH endonuclease but required the helicase UvrD, suggesting that heteroduplexes were recognized by MutS and MutL and then unwound [15].

**Figure 2:**
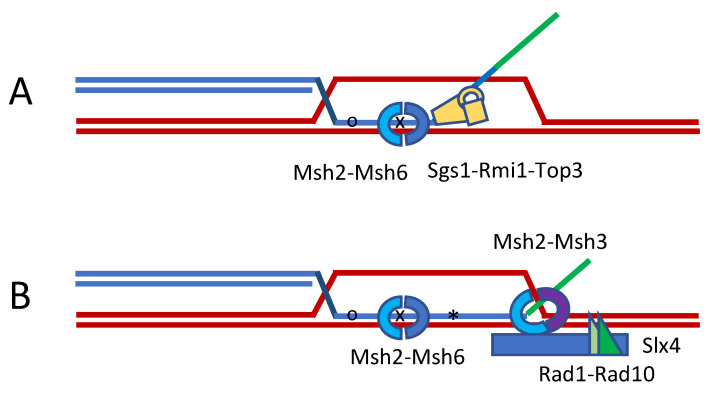
Heteroduplex rejection during strand invasion. The formation of heteroduplex DNA during strand invasion or in SSA will stimulate its unwinding. (**A**) This anti-recombination mechanism is greatly stimulated by the presence of a nonhomologous 3′ tail, which attracts Slx4 and Msh2-Msh3 to enhance the tail’s removal by Rad1-Rad10 (**B**). Recognition of the mismatches requires Msh2-Msh6 and is unwound by the 3′ to 5′ helicase Sgs1 (BLM)-Rmi1-Top3. Heteroduplex rejection does not require MutL homologs, but these are needed to repair the mismatches.

In budding yeast, heterologous substrates are also less efficient in recombination than fully homologous sequences, both in meiotic and mitotic cells [16]. We studied heteroduplex rejection in a Rad51-independent assay, involving single-strand annealing (SSA) [17] (Figure 2). With 3% divergence, SSA was less than 50% efficient; but this barrier could be overcome by deleting the MutS homolog, Msh6. Similarly, repair was restored by a separation-of-function mutation in Msh2 that did not recognize single base pair mismatches but still allowed Msh2 to participate in the removal of nonhomologous single-strand DNA tails that are created during SSA [18]. Tail removal requires not only the Rad1-Rad10 endonuclease that is also needed in nucleotide excision repair, but also Msh2 and Msh3, which apparently recognize and stabilize 3′ flap structures attacked by Rad1-Rad10; moreover, clipping needs the scaffold protein, Slx4 and Saw1 [18,19,20]. So, in this process, Msh2 must associate both with Msh6 and with Msh3 and indeed the competition between rejection and tail-clipping can be tilted by overexpressing Msh6 [17,21].

Unlike the heteroduplex rejection process in *E. coli*, this discouragement of strand annealing is independent of the MutL homologs, Mlh1 and Pms1 [17]. However, the subsequent correction of mismatches in the hetDNA still needs the MutL homologs; in their absence, a colony contains both parental alleles.

In addition, heteroduplex rejection requires the BLM helicase homolog, Sgs1, and its associated proteins Rmi1 and Top3 [17,21]. Sgs1-Rmi1-Top3 were discovered to be a double-Holliday junction “dissolvase”, in which Top3 was needed to resolve the linked strands [22,23]. However, in SSA, Rmi1 and Top3 are still required, perhaps because unwinding starts at both ends and their resolution still needs TopIIIα topoisomerase activity or perhaps simply because Top3 is an essential structural element. Sgs1’s role in heteroduplex rejection has also been well-documented for spontaneous recombination between homeologous sequences [16].

But the 3′-ended nonhomologous tail at the end of heteroduplex DNA proves to be more than a nuisance; it is an essential part of heteroduplex rejection. We constructed a series of strains that can repair an HO-induced DSB by break-induced replication (BIR). We designed these strains so that all the homology shared between the DSB end and the repair template occurred within a 108-bp synthetic intron and in which there was a perfect match between the DSB end and the donor [24]. As a control, we used Cas9 to generate ends with as much as a 34-bp nonhomologous end. With the tailed strain, even a single mismatch in the 108-bp region was sufficient to reduce BIR to about 50% of the level seen in a *msh2*∆ derivative; but in the tailless strain, there was no reduction compared to *msh2*∆ even when every 9th base was mismatched. We have confirmed these results by creating strains in which SSA occurs between 3% mismatched flanking sequences either with long honhomologous tails or such that cleavage occurred between two directly repeated sequences; again, the tailless strain was insensitive to the presence of mismatches (E. Sapède and J.E.H., MS in preparation). How the tail plays such a critical role in recruiting the STR complex and links this to Msh2-Msh6 that detects the mismatches will be important to figure out.

We should not lose sight of the key insight by Radman’s lab that sequence divergence plays a central role in speciation, by preventing recombination between “cousins” as they evolve away from each other [11,12,13]. This is also the case in eukaryotes, as crosses between highly divergent strains—though still both *Saccharomyces cerevisiae*—display marked inviability in meiosis; but this barrier can be greatly reduced by disabling mismatch repair [25].

## 3. BIR and E-SDSA

One of the most stunning instances of DSB repair is the ability of *D. radiodurans* to reassemble its genome after astonishingly high levels of ionizing radiation [26,27]. Radman and his colleagues showed that the restitution depended on homologous recombination and on the fact that *D. radiodurans* carries multiple copies of its genome (it would be impossible to stitch together a shattered genome from a single copy). They considered a number of possible mechanisms that would allow the restitution of an intact genome and concluded that repair would demand a combination of strand invasion between a resected end and a homologous segment, followed by new DNA synthesis, then dissociation of the newly copied DNA and then either another round of invasion/synthesis with another fragmented template or the annealing of two such extended, newly copied DNAs. From the perspective of 2021, the distinction between an incomplete synthesis-dependent strand annealing (SDSA) event and a BIR event has become more difficult to articulate, especially because we have shown that SDSA and BIR are alternative outcomes of a common initiation event in budding yeast [28]. How often second-end capture occurs (for the completion of SDSA) or fails (resulting in BIR) depends on a number of factors. For example, two 3′ to 5′ helicases, Sgs1 (BLM) and Mph1 (FANCM) play opposing roles in assuring second-end capture: while *mph1*∆ drives nearly all the events into BIR, *sgs1*∆ results in almost all outcomes as gene conversions through SDSA. Likely, Sgs1 promotes heteroduplex rejection when second-end capture is attempted, even if there are no mismatches.

The efficiency of second-end capture is also dependent on the strand-annealing functions of Rad52 and its cousin, Rad59 [29]. If the region within the donor sequence that is required for second-end annealing indeed harbors mismatches, the frequency of BIR also goes up significantly at the cost of gene conversion (M.F. Afreen, R. Anand and J.E.H., unpublished).

At least in ectopic recombination, second-end capture may also involve a nonhomologous extension if the region copied from the donor goes past the limits of shared homology; such extensions would need to be clipped off.

**Figure 3:**
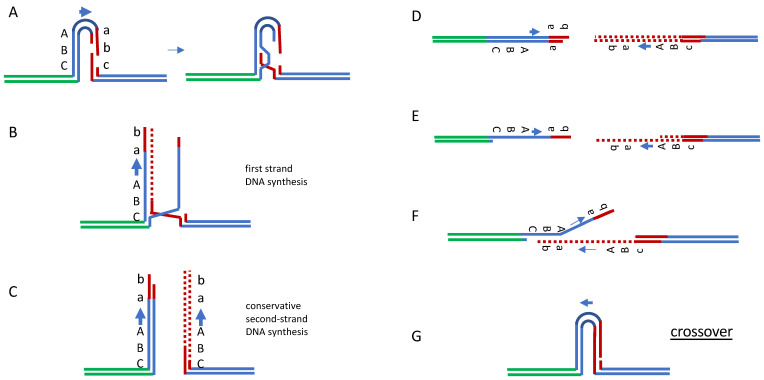
Rad51-independent repair by break-induced replication and single-strand annealing. Extended SDSA can be modeled in yeast by recombination between inverted repeated sequences in which one repeat suffers a DSB. Break-indued replication (**A**–**C**), followed by resection of the ends (**D**,**E**) and SSA (**F**), will complete repair that can result either in a crossover (shown, (**G**)) or noncrossover with equal probability.

A model for the events of E-SDSA (named BIR-SSA in yeast [30,31]) involves the repair of inverted repeats which there is a DSB in one of two repeats sitting in inverted orientation [32] or on a chromosome (Figure 3). When the extent of shared homology is only 33 bp on either side of an HO-induced DSB, Rad51 actually impairs repair; apparently, the amount of homology within the filament is too short to effectuate strand invasion [32]. In the absence of Rad51, repair by BIR-SSA is surprisingly robust, but now dependent on several recombination factors not required for Rad51-dependent gene conversion, including Rad59, Rdh54/Tid1, and Pol32, a non-essential subunit of DNA polymerase δ. In Dienococcus, there is also a RecA-independent aspect to restoring genome integrity [33].

Though hardly in the same league as *D. radiodurans*, budding yeast is actually quite adept in repairing a heavily irradiated diploid. Ref. [34] introduced about 250 DSBs into a diploid cell arrested in G2. Thus among 32 chromosomes, with 4 chromatids, there were about 2 breaks per average-sized chromosome. Survival was measured to be 7% and 28% in two separate experiments. About 2% of the repair events resulted in chromosome aberrations. It would be interesting to determine what proportion of these events required BIR and SSA.

## 4. SOS

And then of course there’s the SOS response to DNA damage. Miro’s ideas shaped everyone’s thinking about the inducibility of DNA repair functions and the cellular responses to damage [35,36]. Yeast is exquisitely sensitive to DNA damage since a single DSB will trigger prolonged checkpoint-mediated arrest. Miro’s ideas as well as those of Evelyn Witkin [37,38] prompted Weinert and Hartwell [39,40,41] and Elledge [42,43] to identify the key elements of the DNA damage checkpoint in budding yeast. Budding yeast will arrest prior to mitosis in response to a single HO-induced DSB but then will adapt after 12–15 h; but two DSBs were sufficient to prevent cell cycle progression entirely [44,45]. The DNA damage checkpoint, governed by the ATR and ATM kinases, results in the induction of many genes and the post-translational modification of many proteins involved in DSB repair [46].

And as with *E. coli*, damage-induced DNA repair in budding yeast is surprisingly mutagenic; recombination-associated DNA repair exhibits a 1000-fold increase in mutations in the newly-copied sequences compared to normal replication [47,48]. However, different from *E. coli*, where much of the mutagenic repair can be attributed to error-prone alternative DNA polymerases, in yeast, the same polymerases, but acting outside the reassuring confines of the intact replication fork—most notably DNA polymerase δ—are responsible for the increased error rate. In contrast, translesion DNA polymerases appear to play only a minor role during DSB repair. Many of the errors suggest that the DNA polymerase frequently dissociates from its template and results in -1 frameshifts in homonucleotide runs, quasipalindrome mutations, and template jumps to homeologous sequences even on different chromosomes [47,49]. These events are also largely independent of the mismatch repair machinery. We speculate that Msh/Mlh proteins normally track behind the replication fork and correct newly generated errors [50], but in repair DNA synthesis this intimate association appears to be lost.

## 5. Summing up

Miro Radman’s contributions to our understanding of DNA repair and mutagenesis have spanned 4 decades. As experimental techniques became more and more precise, Miro’s insights have gone deeper and deeper. We honor his longevity and his passion.

## Data Availability

All data mentioned in this article are published or available from the author.
